# Effects of Fatigue on Balance and Mobility in Subjects with Multiple Sclerosis: A Brief Report

**DOI:** 10.5402/2012/316097

**Published:** 2012-10-31

**Authors:** Elisa Gervasoni, Davide Cattaneo, Angelo Montesano, Johanna Jonsdottir

**Affiliations:** LaRiCE: Gait and Balance Disorders Laboratory, Department of Neurorehabilitation, Don Gnocchi Foundation I.R.C.C.S. Via Capecelatro 66, 20148 Milano, Italy

## Abstract

*Purpose.* People with Multiple Sclerosis (PwMS) tent to have increased levels of fatigue which can impact on their balance and increase risk of falls. However, the relationship between fatigue and balance is poorly understood. The aim of the present study was to assess if an experimentally induced fatigue had an immediate effect on balance. *Methods.* 37 inpatients with multiple sclerosis were recruited; the mean age (standard deviation) was 48.7 (9.6) years. The average onset of the pathology was 15.3 (9.8) years before the start of the study. The median (1°–3° quartile) Expanded Disability Status Scale (EDSS) score was 5.5 (4.5–6.0). Before and after a fatiguing treadmill, session, subjects were assessed with the Berg Balance Scale and Dynamic Gait Index. *Results.* After the treadmill, no statistically significant differences were found in balance before and after a treadmill session (monopodalic stance: before 5.3s (10.3) and after 7.7s (13.9); walk with horizontal head turns: before 11.6 (6.9) seconds and after 11.3 (7.7)). There was no correlation between the EDSS score and the difference in balance skills before and after treadmill. *Conclusion.* After treadmil PwMS were mentally and physically fatigued; however, their balance performance did not change, indicating no increase in risk of falling with fatigue.

## 1. Introduction

Historically, it was believed that fatigue reduced energy for activities of daily living (ADL) in people with multiple sclerosis (PwMS), consequently they were advised to not practice exercise to avoid fatigue [1]. It has also been suggested that fatigue is a fall risk factor implying that increased fatigue during daily life activities would increase the probability of falling [2, 3]. The notion that fatigue could impact negatively on ADL and increase fall risk [2] could again prevent clinicians in suggesting exercise activities to PwMS with balance disorders and symptoms of fatigue. It is important to note that the copresence of fatigue and balance disorders is quite high since 63% of PwMS are fallers [2] and 65% of PwMS report fatigue during ADL [4]. 

Unfortunately, the causal relationship between fatigue and balance is poorly understood. Frozvic [5] and Morris [6] failed to show a decrease of static and dynamic balance from morning to afternoon even if PwMS reported increased fatigue at the end of the day. However these findings could be due to the fact that fatigue was not experimentally induced but only self reported. 

Treadmill has been used to assess walking skills [7] and as an intervention for neurological conditions since it is a highly repetitive form of gait training [8]. It can also be used to experimentally induce fatigue.

The aim of the present study was to assess if experimentally induced fatigue on treadmill would increase balance disorders and consequently fall risk. 

## 2. Method

A convenient sample of 37 PwMS (18 males) was recruited. Inclusion criteria were the ability to maintain upright position at least for 1 minute without support; ability to walk for 6 meters even with assistive device; no history of cardiovascular, pulmonary, and metabolic or other medical conditions.

The study was approved by the ethics review board and the informed consent was obtained for all participants.

To describe the sample the Expanded Disability Status Score (EDSS) [9], the Berg Balance Scale (BBS) [10], the Dynamic Gait Index (DGI) [10], and the Fatigue Severity Scale (FSS) [11] were collected before the treadmill sessions. 

To assess the effect of fatigue on balance, the subjects were tested before and after a single treadmill session. A familiarization phase was carried out to make the subject confident with the task (walking while holding onto the handrail). Speed and inclination were then increased until the subject reported the impossibility of continuing. The day after an experimental treadmill session was carried out at the maximum speed and inclination reached in the familiarization phase until subject reported impossibility to keep walking due to a high level of perceived fatigue. The Rate of Perceived Exertion Scale (RPE or Borg Scale) [12] and heart rate (HR) were monitored at the end of treadmill exercise to assess perceived and physiological domains of fatigue.

To assess variation in balance performances before and after treadmill, the following items of BBS and DGI were tested and timed. BBS: item 11 (turn), 13 (tandem), and 14 (monopodalic stance); DGI: item 1 (6-meters walk), 3 (walk turning head), 4 (walking with head up and down), and 8 (stairs).

Descriptive statistics and box plots were used to detect the presence of outliers. One subjects had abnormal scores and was removed from the analysis. Wilcoxon test was used to assess statistically significant differences between assessments. *P* value for multiple comparison was set at 0.01.

To assess if subjects with higher level of disability had bigger decrement of performance after exercise, we calculated the Spearman correlation coefficient between EDSS score and variation of balance performance of BBS timed items (BBS post-pre).

## 3. Results

The mean age and (standard deviation) of the subjects was 48.7 (9.6) years, 18 were male. The average onset of the pathology was 15.3 (9.8) years before the start of the study. The median (1°–3° quartile) EDSS score was 5.5 (4.5–6.0). Eighteen (48%), 7 (19%), 12 (33%) subjects had a relapsing remitting, a primary progressive, and a secondary progressive subtypes of MS, respectively. The sample showed moderate balance disorders and fatigue; test scores were (median and 2°-3° quartile); 47 (42–51); 15 (12–20); 5.5 (4.9–6.2), respectively, for BBS, DGI, FSS. Subjects walked on the treadmill at 2.5 Km/h for an average of 4.50 (2.05) minutes before they had to stop walking because they had reached maximum exertion. After the treadmill session subjects showed an increase of physiological and perceived fatigue: the mean heart rate was 109.8 beat/min (14.92) and mean RPE score was 16.11 (1.93) corresponding to “hard exercise.”

The effects of fatigue with respect to timed single tasks of BSS and DGI are reported in Table 1; no statistically and clinically significant differences were found before and after treadmill session.

**Figure 1:**
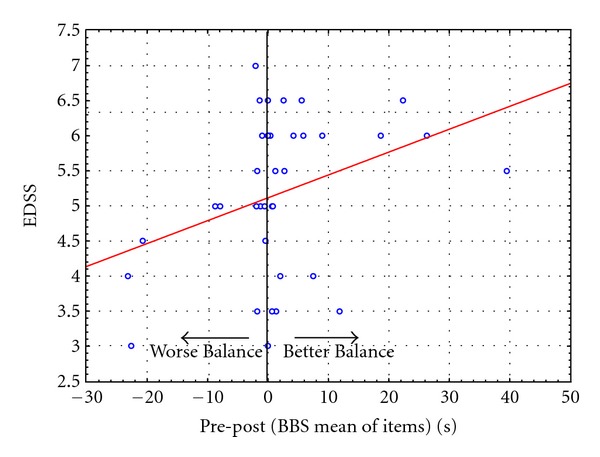
Scatter-plot of EDSS score versus difference in balance skills before and after treadmill. EDSS: Expanded Disability Status Scale; static balance: mean difference scores (in seconds) of timed BBS items (after treadmill-before treadmill).

There was no correlation (*r* = 0.28, *P* > 0.05) between EDSS and variation of balance performance after treadmill (Figure 1).

## 4. Discussion

Our results did not support the hypothesis that fatigue increases balance disorders and ultimately fall risks in PwMS.

In this study fatigue was experimentally induced using walking, a typical task involved in daily life activities. Right after the treadmill section MS subjects had a high level of exertion as demonstrated by an abnormal increase of RPE (up to 16 points) with a HR of 110 beat/min. This rate of exertion can be considered abnormal since normative data on healthy subjects indicates that exercising at 110 beat/min would correspond to a score of 11 points on the RPE [13].

The objective was to bring the subjects to max exertion as measured by perceived effort. The treadmill speed was set immediately at the max speed reached by the subjects in the previous familiarization session and they were urged to walk as far as they could. What was surprising was how fast they arrived at max exertion, on the average they walked only 4 minutes before arriving at the maximum exertion. The mean speed was 2.5 km/h but with a range from 1 to 4.5. This highlights difficulties PwMS may have in carrying out activities of daily living in the community where walking distances easily “go beyond” the 4-5 minutes.

In our sample all demonstrated fatigue and balance disorders but contrary to our expectations, there was no significant decrement in balance after treadmill. This implies that even if subjects reported being very tired, their balance performance remained unchanged; thus, since BBS is a fall predictor [14] no increase of fall risk factor is expected with increase in fatigue. Neither was there an effect of fatigue on walking performance; subjects walked at the same speed even during more challenging tasks (e.g., walking with head turn). The present study findings support observational studies reporting no decrement in balance performance when subjects are fatigued [5, 6, 16]. A recent study by Van Emmerick and colleagues [15] similarly found no impact of muscle fatigue on balance in less challenging balance conditions, although they found differences in more challenging conditions. However, their fatiguing procedure differed from that used in the present study which might explain these differences in results. 

In addition, our subjects had on the average an EDSS of 5.5 and so they were relatively compromised in the walking abilities which may explain difference between the results of the present study and others that assessed a less compromised population [17].

Finally, no correlation between disability level and variation in balance performance after fatigue was observed suggesting that also subjects with higher levels of disability did not show balance deterioration after treadmill. 

The main limits of this pilot study are the relatively small number of patients, and the relatively short time of exercise. Further, measuring oxygen uptake during exertion and instrumental measures of balance might add information on the effect of intense exercise on fatigue and balance.

In conclusion our results suggest that PwMS should be encouraged to exercise and lead an active lifestyle since exercise may improve their emotional and physical status without however deteriorating balance and increasing risk of falling. 

## Figures and Tables

**Table 1:** Static and dynamic balance performance in some BBS and DGI items before and after treadmill session.

	*N*	Pre	Post	Post-Pre	*P* value
Turn	37	5.7 (2.7)	5.6 (2.7)	−0.1^*∧*^	0.56
Tandem	37	25.5 (22.3)	26.8 (22.0)	1.4^*∧*^	0.62
Monopodalic stance	37	5.3 (10.3)	7.7 (13.9)	2.3^*∧*^	0.04
6-metres walk	37	9.5 (4.9)	10.0 (8.2)	0.5*	0.47
Walk (turning head)	37	11.6 (6.9)	11.3 (7.7)	−0.3^*∧*^	0.80
Walk (head up and down)	37	10.7 (5.5)	10.8 (6.9)	0.1*	0.34
Stairs	37	12.7 (6.9)	13.0 (7.0)	0.3*	0.76

Mean and (standard deviation) are reported in seconds. Note an *indicate decrement of performance, while ^*∧*^indicate improvement of performance.
